# 髓系肿瘤合并坏疽性脓皮病6例报告并文献复习

**DOI:** 10.3760/cma.j.issn.0253-2727.2023.11.011

**Published:** 2023-11

**Authors:** 晶晶 俞, 丽亚 马, 伟来 徐, 琛 梅, 歆平 周, 丽 叶, 红艳 佟

**Affiliations:** 1 浙江大学附属第一医院血液科，杭州 310058 Department of Hematology, The First Affiliated Hospital of Zhejiang University, Hangzhou 310058, China; 2 浙江大学附属第一医院象山分院血液科，宁波 310058 Department of Hematology, Xiangshan Hospital of the First Affiliated Hospital of Zhejiang University, Ningbo 315700, China; 3 浙江省人民医院血液科，杭州 310014 Department of Hematology, Zhejiang Provincial People's Hospital, Hangzhou 310014, China

坏疽性脓皮病（Pyoderma gangrenosum，PG）由Brunsting、Goeckerman和O'Leary于1930年首次报道[1]–[2]。典型的PG表现常有快速进展的疼痛性的坏死性溃疡，通常需要进行皮肤活检等以排除其他疾病。PG合并最常见的原发病依次为炎症性肠病、关节炎、血液系统疾病。血液系统疾病作为PG排名前三的合并症，国内多为个案报道[3]–[4]。本文我们回顾分析我院收治的髓系肿瘤合并坏疽性脓皮病6例，结合国内外新近文献，总结其临床特点和治疗反应如下。

## 病例资料

浙江大学附属第一医院血液科自2014年至今共收治髓系肿瘤合并PG患者6例，其中5例经病理活检证实，符合Su等[1]提出的诊断标准或Maverakis等[2]提出的替代性诊断标准；1例虽未经活检，但仍符合文献[1]标准。患者中位年龄44（36～64）岁，男女各3例。合并骨髓增生异常综合征（MDS）4例，合并原发性骨髓纤维化（PMF）2例。其中3例在确诊血液病基础上发生PG，2例在诊断PG后发现血液病，1例（例5）同时发现血液病和PG。病例临床特征见表1，皮肤损害分布范围广泛，其中4例为多个部位累及，累及头面部2例、上肢2例、下肢3例、肛周1例。皮肤损害均表现为由微小的红斑或者水疱迅速进展至局部肿胀、糜烂、破溃，部分结痂（图1），病程中均伴有皮损部位疼痛。5例病程中合并高热，均予经验性抗感染治疗，4例无效、另1例体温恢复正常但皮损继续进展。6例患者均合并不同程度的贫血，1例PMF患者WBC正常，另1例PMF及4例MDS均存在不同程度的WBC减低。其中5例行皮肤活检，病理均提示为皮肤组织慢性化脓性炎或中性粒细胞浸润（图2），均未行中性粒细胞克隆性检测。坏死部位培养出肺炎克雷伯菌1例，大肠埃希菌1例。治疗过程及疗效：初均予抗感染治疗，对皮损无效，其中1例在诊断明确前经历多次清创及植皮，但皮损仍扩大。所有患者加用糖皮质激素治疗后，均在2～10 d内出现不同程度的缓解。1例合并PMF患者糖皮质激素治疗早期有效，但治疗1个月后皮损再次扩大，未缓解。余5例均在2周内皮损愈合，其中3例在糖皮质激素逐渐减量后未见PG复发；2例在糖皮质激素减量2～3个月后轻度复发，予加量后再次好转。

**图1:**
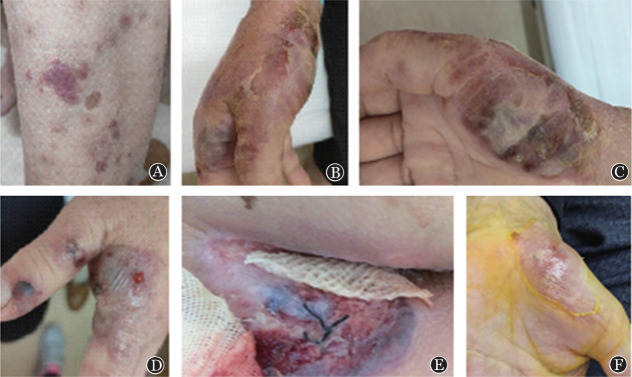
髓系肿瘤合并坏疽性脓皮病患者的皮肤损害表现 A 新发的位于小腿前方的紫红色结节，边界不规则； B~D 囊泡表面坏死和浅层糜烂，边界紫红色、被红斑和水肿包围，部分结痂； E 肛周皮肤溃疡坏死，边界呈红-蓝色，潜行性破坏； F 皮肤遗留皱纹纸样疤痕

**图2:**
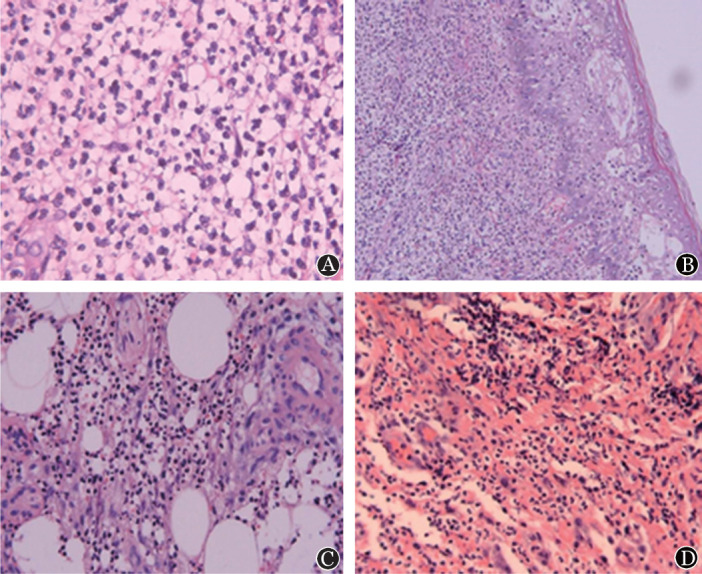
髓系肿瘤合并坏疽性脓皮病患者皮肤活检的病理表现（HE染色） A 棘层肥厚，真皮浅层水肿、中性粒细胞浸润（高倍）； B 皮肤慢性化脓性炎伴大量中性粒细胞浸润（低倍）； C 真皮层慢性化脓性炎伴鳞状上皮增生及轻度异型增生（中倍）； D 表皮局部缺损，真皮层弥漫炎细胞浸润，可见较多中性粒细胞（中倍）

**表1:** 6例髓系肿瘤合并坏疽性脓皮病（PG）患者皮疹的临床特征及治疗过程

例号	原发血液病	皮疹表现	病理结果	PG相关治疗及转归	血液病治疗及转归
1	PMF	肛周皮肤糜烂化脓，表面糜烂破溃，触痛明显，边界呈红-蓝色	棘层肥厚，真皮浅层水肿、中性粒细胞浸润	抗感染及局部换药，未缓解；加用甲泼尼龙40 mg/d静脉治疗2 d后渗出及疼痛好转，11 d后肛周溃疡基本吸收；14 d后改甲泼尼龙口服并逐渐减量	PMF未治疗，4个月后出现淋巴瘤；治疗无效死亡，总病程5个月
2	PMF	左前臂留置针旁皮肤溃烂肿胀，伴疼痛；口角及颊部肿胀破溃渗出疼痛伴脓性水疱，伴黑痂；双上肢多发溃疡	皮肤慢性化脓性炎伴大量中性粒细胞浸润	抗感染治疗无好转，3 d后加甲泼尼龙40 mg/d静脉治疗，第9天上肢肿胀消退、部分结痂、逐渐减量，第18天出现口角及面颊部皮损，改甲泼尼龙40 mg每12 h 1次，仍加重	疾病进展，放弃治疗。初诊发现到放弃治疗共4个月
3	MDS（IPSS低危）	双下肢皮肤圆形红斑隆起，逐渐增大融合，表面浅溃疡，可见血性及脓性分泌物，边缘不规则，部分结痂，伴疼痛	真皮层慢性化脓性炎伴鳞状上皮增生及轻度异型增生	初予广谱抗感染无好转；第8天予甲泼尼龙40 mg/d，第14天皮损好转。3个月后减量为泼尼松10 mg/d，右下肢皮损复发伴疼痛高热，再次甲泼尼龙40 mg/d，逐渐减量未复发	MDS为低危，予观察随访1年无进展，1年后失访
4	MDS（IPSS高危）	颈部左侧水疱，直径约0.5 cm，逐渐加重、蔓延，伴头面部、颈部左侧红肿，部分水疱破溃，可见脓性分泌物渗出，伴疼痛	表皮局部缺损，真皮层弥漫炎细胞浸润，可见较多中性粒细胞	广谱抗感染，疼痛及皮损加重；第11天加用地塞米松15 mg/d，次日疼痛好转，并逐渐减量，第14天皮肤破溃处结痂，未复发	MDS 10年未治疗，发现PG后1年进展为AML-M_5_，先后予IA、HA、CAG方案化疗，发现白血病6个月后因骨髓抑制伴严重感染死亡
5	MDS（IPSS高危）	四肢皮肤散在红色皮疹，大小不一，随后出现双手红肿，皮疹面积及范围扩大，互相融合，伴破溃流液，四肢多发新旧不一的紫红色丘疹及疱疹，部分巨大疱疹伴结痂	未做	次日起甲泼尼龙40 mg每12 h 1次静脉治疗，第5天皮疹好转，第8天皮损消退，遗留皱纹纸样疤痕，第9天起改口服并逐渐减量，未复发	地西他滨治疗6个月后完全缓解，停药12个月后复发，继续地西他滨治疗24个月后转为AML，维奈克拉+HAG化疗后缓解，45个月后失访
6	MDS（IPSS中危-2）	右大腿后侧皮肤红肿坏死，中央色黑，逐渐增大，伴皮肤破溃及坏死，疼痛明显，表面部分结痂，部分为皮肤缺损的暗红色创面，可见明显的皮肤坏死组织，渗出明显，边界欠清，创面周缘可见明显红肿	表皮及真皮内大量中性粒细胞、多核细胞浸润，真皮内血管扩张，出血，毛细血管增生	初期予多次清创、换药、抗感染、输血对症支持治疗1个月余，仍反复高热、溃疡皮损面扩大；加用泼尼松20 mg每日2次，次日起体温正常，2周内溃疡逐渐缩小至愈合；泼尼松减量2个月后少量皮疹复发（未发生溃疡坏死）	因患方原因，MDS未治疗未复查

注 PMF：原发性骨髓纤维化；MDS：骨髓增生异常综合征；IPSS：国际预后积分系统；AML：急性髓系白血病

## 讨论及文献复习

PG是一种非感染性嗜中性粒细胞性皮病（Neutrophilic dermatoses），其年发病率约0.63/10万[5]，发病机制尚不完全明确，可能与G-CSF、IL-8的表达改变、中性粒细胞功能异常和遗传易感性有关[6]–[8]。2007年，Magro等[9]在皮肤活检标本中发现了克隆性浸润的证据，且无论有无骨髓增生性疾病（各7例），患者中克隆性浸润发生率相同（各5例，占81％）。2013年，Sujobert等[10]使用荧光原位杂交技术证实嗜中性皮肤病中浸润性中性粒细胞与潜在的髓系肿瘤克隆相关，推测嗜中性皮肤病可能是潜在的髓系肿瘤分化异常的一种表现。

PG常伴有潜在的系统疾病，既往认为最常见的合并症依次为炎症性肠病、关节炎、血液系统疾病[1],[7]，与PG相关的血液系统疾病的发生率为3.9％～20.0％[7],[11]，但德国Al Ghazal等[12]资料显示血液系统疾病更常见，贫血发生率高达45.6％。Montagnon等[13]统计了文献报道中所有符合诊断条件的血液系统恶性肿瘤相关的PG病例，发现MDS最为常见；国内吴超等[14]分析了北京协和医院近20年间收治的61例PG患者，血液病最常见（10例，16.39％），其中MDS 4例。本组6例髓系肿瘤合并PG，其中4例为MDS，2例为PMF。髓系肿瘤易合并PG的原因不明，亦有不少患者可于PG后确诊或与PG同时诊断，故对于诊断为PG的患者需警惕血液系统疾病尤其是髓系肿瘤可能，这些患者往往存在血常规异常，需要进行系统检查。

PG的诊断依靠临床特征和病理活检，病理活检只需证实存在中性粒细胞浸润，无需克隆性检测标准。皮损累及部位以四肢、头面部和肛周多见。皮损表现为由微小的红斑或者水疱迅速进展至局部肿胀、糜烂、破溃，部分结痂、坏死。皮损部位可以继发感染，但皮损对抗感染治疗一般无效。本组2例患者在皮损部位培养出细菌，抗感染治疗无效。

PG的有效治疗药物为糖皮质激素和环孢素A，国外推荐治疗剂量为泼尼松0.5～1.0 mg·kg^−1^·d^−1^[15]。Ormerod等[16]对121例临床诊断PG的患者进行多中心平行组观察者盲随机对照研究，结果显示甲泼尼龙0.75 mg·kg^−1^·d^−1^和环孢素A 4 mg·kg^−1^·d^−1^疗效及安全性差异均无统计学意义。国内冯尘尘等[17]的综述中提到目前PG的治疗主要为糖皮质激素、免疫抑制剂和生物制剂等。本组6例患者均对糖皮质激素（甲泼尼龙40～80 mg/d或等效剂量）治疗有效，但减量过程中有复发。其中例5经去甲基化治疗MDS得到完全缓解后，PG长期未复发。

Langan等[5]的一项回顾性队列研究表明，PG患者和3组与年龄、性别和实际病情相匹配的对照组（普通人群，类风湿关节炎患者和炎症性肠病患者）比较，PG的死亡风险是其他对照组的3倍。血液病患者合并PG更容易向恶性转化[12]–[13]，本组6例中3例短期内因原发病进展而死亡。

综上，PG是一种非感染性嗜中性粒细胞性皮肤病，其诊断依靠临床表现和病理活检。糖皮质激素是其有效治疗药物。MDS、PMF等髓系肿瘤易合并PG，合并PG常导致血液病预后不良，更容易向恶性转化。在PG得到有效控制下，要及时对血液系统原发病进行治疗。若原发病好转，PG可达到更好的控制及减少复发，但因病例数量少，仍需验证。
